# Mesenchymal Stem Cell-Based Therapy of Inflammatory Lung Diseases: Current Understanding and Future Perspectives

**DOI:** 10.1155/2019/4236973

**Published:** 2019-05-02

**Authors:** C. Randall Harrell, Ruxana Sadikot, Jose Pascual, Crissy Fellabaum, Marina Gazdic Jankovic, Nemanja Jovicic, Valentin Djonov, Nebojsa Arsenijevic, Vladislav Volarevic

**Affiliations:** ^1^Regenerative Processing Plant, LLC, 34176 UD Highway 19 N Palm Harbor, Palm Harbor, Florida, USA; ^2^Emory University School of Medicine, 648 Pierce Dr NE, Atlanta, GA, USA; ^3^Atlanta VA Medical Center, 1670 Clairmont Rd, Decatur, Atlanta, GA, USA; ^4^Department of Genetics, Faculty of Medical Sciences, University of Kragujevac, Serbia; ^5^Center for Molecular Medicine and Stem Cell Research, Faculty of Medical Sciences, University of Kragujevac, 69 Svetozar Markovic Street, Kragujevac, Serbia; ^6^Institute of Anatomy, University of Bern, 2 Baltzerstrasse, Switzerland; ^7^West Pasco Pulmonary Associates, 7545 Medical Dr, Hudson, Florida, USA

## Abstract

During acute or chronic lung injury, inappropriate immune response and/or aberrant repair process causes irreversible damage in lung tissue and most usually results in the development of fibrosis followed by decline in lung function. Inhaled corticosteroids and other anti-inflammatory drugs are very effective in patients with inflammatory lung disorders, but their long-term use is associated with severe side effects. Accordingly, new therapeutic agents that will attenuate ongoing inflammation and, at the same time, promote regeneration of injured alveolar epithelial cells are urgently needed. Mesenchymal stem cells (MSCs) are able to modulate proliferation, activation, and effector function of all immune cells that play an important role in the pathogenesis of acute and chronic inflammatory lung diseases. In addition to the suppression of lung-infiltrated immune cells, MSCs have potential to differentiate into alveolar epithelial cells *in vitro* and, accordingly, represent new players in cell-based therapy of inflammatory lung disorders. In this review article, we described molecular mechanisms involved in MSC-based therapy of acute and chronic pulmonary diseases and emphasized current knowledge and future perspectives related to the therapeutic application of MSCs in patients suffering from acute respiratory distress syndrome, pneumonia, asthma, chronic obstructive pulmonary diseases, and idiopathic pulmonary fibrosis.

## 1. Introduction

The respiratory system is continuously *exposed* to various irritants such as inhaled toxins, carbon granules, pathogens, and their products. Pulmonary homeostasis is maintained by interaction between alveolar epithelial cells and lung-resident immune cells that continually monitor the pulmonary microenvironment, induce tolerance to innocuous inhaled particles, or provide efficient immune reactions against invading microbes [1]. Accordingly, in the lungs, inflammation is the result of the infection, trauma, and hypersensitivity caused by pathogens, airborne irritants, hazardous pollutants, toxins, and allergens. Pathogen-associated molecular patterns (PAMPs) expressed on the lung infiltrated microbes, as well as damage-associated molecular patterns (DAMPs) and alarmins, released from the injured lung parenchymal cells, activate residential macrophages which produce a large amount of inflammatory chemokines and cytokines, attract circulating immune cells in the lungs, and initiate inflammation. Clinically, acute lung injury and inflammation is seen in pneumonia and acute respiratory distress syndrome (ARDS), whereas chronic inflammation is represented by asthma and chronic obstructive pulmonary diseases (COPD) [2]. The repair of the airway epithelium after acute or chronic injury is modulated by matrix metalloproteinases (MMPs), cytokines, and growth factors produced by epithelial cells, lung-resident immune cells, fibroblasts, and chondrocytes [1]. Inappropriate immune responses and/or aberrant repair process causes irreversible damage in lung tissue and most usually results in the development of fibrosis followed by decline in lung function [3]. Inhaled corticosteroids are very effective in patients with inflammatory lung disorders, but their long-term use is associated with an increased risk of pneumonia, oral candidiasis, osteoporosis, skin bruising, and tuberculosis [4]. Accordingly, new therapeutic agents that will attenuate ongoing inflammation and prevent accumulation of fibrous connective tissue on one side and, at the same time, promote regeneration of injured alveolar epithelial cells are urgently needed.

Due to their capacity to suppress detrimental immune response and ability to differentiate into type II alveolar epithelial (ATII) cells *in vitro*, mesenchymal stem cells (MSCs) represent new players in cell-based therapy of acute and chronic inflammatory lung disorders [5, 6]. Since these adult multipotent stem cells can be readily isolated from numerous tissues (bone marrow (BM), adipose tissue (AT), amniotic fluid (AF), placenta (PL), umbilical cord (UC), peripheral blood, lungs, and deciduous teeth) and expanded with high efficiency, MSC-based therapy of lung diseases has rapidly progressed over the past decade [5]. Accordingly, in this review article we summarized findings obtained in preclinical and clinical studies that demonstrated beneficent effects of MSCs in the treatment of lung diseases. An extensive literature review was carried out in July 2018 across several databases (MEDLINE, EMBASE, Google Scholar, and ClinicalTrials.gov), from 1990 to present. Keywords used in the selection were “mesenchymal stem cells,” “inflammatory lung disease,” “ARDS,” “lung injury,” “COPD,” “asthma,” and “idiopathic pulmonary fibrosis (IPF).” Eligible studies had to delineate molecular and cellular mechanisms involved in the MSC-based therapy of acute and chronic inflammatory lung diseases, and their findings were analyzed in this review.

## 2. Main Text

MSCs are self-renewable, multipotent cells capable of suppressing immune response and differentiating into ATII cells *in vitro* [5, 6]. Accordingly, MSC-mediated suppression of inflammation and, at the same time, MSC-dependent lung repair and regeneration were responsible for their therapeutic effects in the treatment of ARDS, pneumonia, asthma, COPD, and IPF.

## 3. Molecular Mechanisms Responsible for MSC-Based Beneficial Effects in the Therapy of Lung Diseases

MSCs are able to modulate proliferation, activation, and effector function of all immune cells that play an important role in the pathogenesis of inflammatory lung diseases, including professional antigen-presenting cells (dendritic cells (DCs), macrophages, and B lymphocytes), neutrophils, and effector and regulatory T cells. MSCs alter immune response through juxtacrine or paracrine mechanisms [7]. MSCs lack the surface expression of costimulatory molecules and are able to render Th1, Th2, and Th17 cells anergic. Additionally, interaction of the inhibitory molecule programmed death 1 (PD-1) with its ligands PD-L1 and PD-L2 was responsible for MSC-mediated inhibition of T cell proliferation [5]. Precisely, upregulation of the cyclin-dependent kinase inhibitor p27kip1 and inhibition of cyclin-D2 were observed in T cells after a cross-talk with MSCs. In this way, transplanted MSCs significantly reduce the total number of effector T cells in the injured lungs and attenuate Th1-, Th2-, or Th17-driven inflammation [5].

In addition to juxtacrine mechanisms, MSCs may suppress ongoing T cell-dependent inflammation through the secretion of soluble, immunosuppressive factors (prostaglandin E2 (PGE2), transforming growth factor beta (TGF-*β*), indoleamine 2,3-dioxygenase (IDO), and nitric oxide (NO)) [8]. Through the production of PGE2, MSCs attenuate the expression of the interleukin- (IL-) 2 receptor and, accordingly, inhibit clonal expansion of activated T cells. TGF-*β* is also a potent inhibitor of the IL-2 signaling pathway and is involved in MSC-mediated G1 cell cycle arrest of activated T cells. In a similar manner, MSC-derived NO inhibits phosphorylation of signal transducer and activator of transcription- (STAT-) 5 in T cells, leading to cell cycle arrest while MSC-derived IDO promotes the degradation of tryptophan into kynurenine which suppresses proliferation or induce apoptosis of activated T cells [8].

In addition to the direct suppression of effector T cells, MSCs are able to suppress the generation of Th1, Th2, and Th17 cells by modulating the antigen-presenting function of DCs in a PGE2-, IL-10-, and IL-6-dependent manner [5]. After an interaction with MSCs, DCs became immature, with a reduced capacity for antigen presentation, due to the reduced expression of the major histocompatibility complex (MHC) and costimulatory molecules. Additionally, MSCs can induce tolerogenic phenotype in DCs and may promote polarization of inflammatory M1 macrophages towards immunosuppressive M2 macrophages. In this way, MSCs reduce the production of inflammatory cytokines (tumor necrosis factor alpha (TNF-*α*), IL-1*β*, and IL-12) in DCs and macrophages and promote the production of anti-inflammatory IL-10 and TGF-*β* resulting in enhanced tissue repair and regeneration [5, 9–13]. In an IL-10- and TGF-*β*-dependent manner, tolerogenic DCs and M2 macrophages induce enhanced production of immunosuppressive human leucocyte antigen- (HLA-) G5 in MSCs and promote their capacity to stimulate generation and expansion of T regulatory cells (Tregs) [14], contributing to the creation of the anti-inflammatory microenvironment in the injured lungs.

In addition to their interaction with lung-infiltrated immune cells, MSCs have potential to differentiate into ATII-like cells *in vitro*. Ma and coworkers provided the first evidence that BM-derived MSCs (BM-MSCs) can be successfully differentiated into ATII cells *in vitro* after coculturing with MRC-5 cells (derived from normal fetal lung mesenchymal tissue) in a modified small airway growth medium (SAGM) that contained bovine serum albumin (BSA, 0.5 mg/ml), insulin (5 mg/ml), transferrin (10 mg/ml), bovine pituitary extract (30 mg/ml), adrenaline (epinephrine, 0.5 mg/ml), fibroblast growth factor (FGF)10 (1ong/ml), cortisol (0.5 mg/ml), human EGF (epidermal growth factor, 0.5 ng/ml), and antibiotics (gentamicin sulfate, 0.05 mg/ml; amphotericin-B, 0.05 mg/ml) [15]. ATII-like cells became dominant in culture two to three weeks after interaction of BM-MSCs with MRC-5, and at the same time point, surfactant protein (SP) C, a specific functional marker of human ATII cells, was detected in differentiated cells [15]. In a similar manner as BM-MSCs, AF-derived MSCs (AF-MSCs) and decidua-derived MSCs (D-MSCs) can be differentiated into ATII cells *in vitro* [16, 17]. Li and coworkers demonstrated that, with the use of the appropriate induction medium, including KnockOut™ serum replacement (KOSR), activin A, and small airway basal medium, AF-MSCs differentiate into SPC-expressing ATII-like cells *in vitro* [16]. Similar findings were reported by Cerrada and colleagues who demonstrated that D-MSCs cultured in the small airway epithelial cell growth medium successfully generated functional ATII-like cells which were able to exocytose lipid-rich assemblies with high surface-active capabilities [17].

Activation of canonical as well as noncanonical Wnt pathways is crucially important for differentiation of MSCs into ATII-like cells [18, 19]. Liu and associates used a modified coculture system with murine lung epithelial-12 (MLE-12) cells and SAGM to demonstrate that Wnt3a-induced activation of the canonical Wnt/*β*-catenin pathway resulted in differentiation of murine MSCs in functional ATII-like cells which expressed specific markers: SPC, SPB, and SPD [18]. Members of the same research group documented that Wnt5a-induced activation of noncanonical Wnt/c-Jun N-terminal kinase (JNK) or Wnt/protein kinase C (PKC) pathways could also result in the successful differentiation of MSCs into ATII-like cells *in vitro* [19]. Furthermore, addition of dickkopf Wnt signaling pathway inhibitor 1 (Dkk1) as well as JNK or PKC inhibitors to the SAGM suppressed the activation of canonical and noncanonical Wnt pathways and completely abrogated the capacity of MSCs to generate ATII-like cells [18, 19]. In line with these results are findings reported by Shi and coworkers who demonstrated that Wnt/*β*-catenin signaling may be an essential mechanism underlying the regulation of epithelial differentiation of lung residential MSCs [20]. Nevertheless, it has to be highlighted that differentiation of MSCs in ATII-like cells was mainly documented *in vitro*, while MSC-dependent beneficial effects in attenuation of inflammatory lung diseases were mainly based on the paracrine effects of transplanted MSCs. Accordingly, future experimental studies must provide stronger evidence about the capacity of MSCs to differentiate into ATII-like cells *in vivo*.

Having in mind that MSCs have the capacity to generate ATII cells and that injury of ATII cells and alveolar-epithelial barrier represents the main pathological characteristic observed in patients suffering from ARDS, several experimental studies investigated the therapeutic potential of MSCs in the treatment of ARDS.

## 4. MSC-Mediated Attenuation of ARDS and Pneumonia

ARDS is a severe clinical syndrome triggered by the disruption of the alveolar-epithelial barrier, accompanied with interstitial edema and infiltration of inflammatory *cell*s that resulted in progressive acute respiratory failure [21–24]. This “exudative phase” is followed by a fibrotic phase characterized by proliferation of type II pneumocytes, fibroblasts, myofibroblasts, and matrix deposition [25]. Although numerous pharmacologic agents, including inhaled synthetic surfactants, ketoconazole, simvastatin, and ibuprofen, have been tested in the therapy of ARDS, none of them managed to significantly reduce a notably high mortality rate of ARDS which remains at 34-44 percent [26].

**Figure 1:**
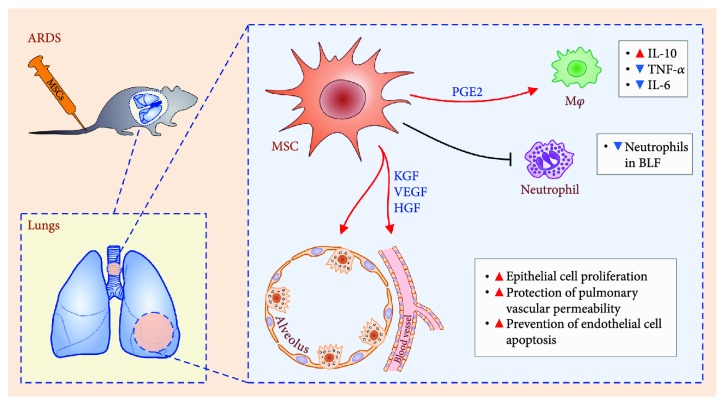
Molecular mechanisms responsible for MSC-based attenuation of ARDS. Intravenously injected MSCs engrafted in the ARDS-injured lungs and, in a paracrine manner (through the production of KGF, VEGF, HGF), promoted proliferation of epithelial cells, induced protection of vascular permeability, and prevented apoptosis of endothelial cells. Additionally, MSC-based therapy reduced the presence of neutrophils in bronchoalveolar lavage fluid (BLF) and in a PGE2-dependent manner suppressed the production of inflammatory cytokines (TNF-*α* and IL-6) and stimulated the secretion of immunosuppressive IL-10 in alveolar macrophages.

Several, recently conducted, preclinical studies demonstrated that MSCs and their secretomes could be considered as new and effective therapeutic agents for the treatment of ARDS (Figure 1). ARDS often develops as a complication of severe sepsis, particularly after infection with Gram-negative bacteria [27]. MSC treatment prevented the development of ARDS in an animal model of sepsis induced by *Escherichia coli*-derived lipopolysaccharide (LPS) [28, 29]. Systemic application of MSCs in a mouse model of LPS–induced ARDS significantly ameliorated alveolar injury and inflammation [30]. MSCs, in a paracrine, IL-10-dependent manner, attenuated the influx of neutrophils in the lungs and decreased the production of inflammatory TNF-*α* in lung-infiltrated immune cells [30, 31]. Additionally, through the production of the keratinocyte growth factor (KGF), vascular endothelial growth factor (VEGF), and hepatocyte growth factor (HGF), MSCs promoted the regeneration of ATII cells, prevented the apoptosis of endothelial cells, and contributed to the enhanced repair of the alveolar-epithelial barrier in the ARDS-injured lungs [32–34]. A diminished inflammatory injury in MSC-treated animals correlated with reduced edema, improved oxygenation, and prolonged survival [28, 29]. Additionally, MSCs protect from sepsis-associated ARDS by increasing the capacity of macrophages to produce anti-inflammatory IL-10 in a PGE2-dependent manner [35].

In addition to their regenerative and immunosuppressive effects responsible for the attenuation of ARDS, MSCs are also able to promote resolution of ongoing inflammation by producing proresolving mediator lipoxin A4 (LXA4) which reduces pulmonary edema and promotes survival of mice suffering from LPS-induced ARDS [36].

Several immunomodulatory factors, released during ARDS, may reduce differentiation of MSCs into ATII-like cells and consequently attenuate therapeutic potential of MSCs in the treatment of ARDS. Overexpressed microRNA-615-3p or microRNA-155-5p inhibits differentiation of MSCs into ATII cells leading to the progression of ARDS [37, 38]. Pathological changes in the microenvironment of ARDS-injured lungs, in turn, negatively affect the capacity of MSCs to proliferate and differentiate into ATII cells [39]. In this way, a negative loop is created that attenuates therapeutic potential of MSCs and contributes to the further progression of acute lung injury.

The therapeutic potential of MSCs in the treatment of ARDS has been evaluated in several, already completed, clinical trials [40, 41]. Wilson and coworkers demonstrated that single intravenous infusion of allogeneic BM-MSCs (1, 5, or 10 million cells/kg of body weight (bw)) was well tolerated in nine patients with moderate to severe ARDS (NCT01775774). Side events, clinical instability, or dose-limiting toxicity was not observed in any of the nine patients that received allogeneic BM-MSCs [40]. Results obtained in this study were used as an optimistic starting point for a larger randomized, multicenter, phase 2 clinical trial that was conducted from 2014 to 2018 in the United States (NCT02097641). The trial enrolled 60 adult ARDS patients who intravenously received either a single dose of allogeneic BM-MSCs (10 million cells/kg bw) or placebo (Plasma-Lyte A). Although this trial has been completed in February 2018, the obtained results are not published yet.

In another clinical study, Zheng and colleagues reported that intravenous administration of allogeneic MSCs is a safe but not efficient therapeutic approach in the treatment of ARDS patients (NCT01902082). Twelve adult patients with ARDS safely received one intravenous dose of allogeneic AT-MSCs (1 million cells/kg bw), but AT-MSC-based therapy did not significantly attenuate serum levels of inflammatory cytokines (IL-6 and IL-8) and did not manage to improve lung function in ARDS patients [41].

ARDS and pneumonia are interrelated in critically ill patients. Pneumonia is considered as the main cause of ARDS while ARDS is usually complicated by nosocomial pneumonia [42]. Heat shock proteins or other DAMPs, released from injured lung parenchymal cells, as well as PAMPs of inhaled pathogens, induce Toll-like receptor- (TLR-) dependent activation of the nuclear factor kappa B (NF-*κ*B) pathway in alveolar macrophages leading to the enhanced secretion of CXCL8 and CXCL11. An increased concentration of these inflammatory chemokines in inflamed lungs attract interferon gamma- (IFN-*γ*-) producing neutrophils and CD4+Th1 cells which, in turn, enhance the secretion of inflammatory cytokines and proteolytic enzymes in alveolar macrophages, creating a “positive inflammatory loop” in the injured lungs [43]. Alveolar macrophages play a crucially important role in the bacterial clearance and attenuation of bacterial pneumonia, the most common infectious cause of death worldwide [44]. It was recently revealed that MSCs produce microvesicles which may promote phagocytic activity of alveolar macrophages resulting in the alleviation of bacterial pneumonia induced by Gram-negative *Escherichia coli* [45]. Additionally, MSCs produce antibacterial proteins and are able to directly suppress bacterial growth in the inflamed lungs [46]. Intratracheal administration of MSCs significantly attenuated lung injury and inflammation and improved the survival of experimental animals with bacterial pneumonia by promoting bacterial clearance in a lipocalin-2-dependent manner [46]. LPS-induced activation of TLR-4 in MSCs enhances secretion of lipocalin-2 that binds bacterial ferric siderophores, reduces the uptake of iron, and suppresses bacterial growth [47]. In line with these findings are results recently reported by Gupta and coworkers who found that mutation of TLR-4 in MSCs significantly impaired their therapeutic efficacy in an experimental model of bacterial pneumonia [48]. Accordingly, intratracheal administration of TLR-4-primed MSCs should be explored in future experimental studies as a potentially new cell-based therapeutic approach for the elimination of antibiotic resistant Gram-negative bacterial strains in the inflamed lungs.

## 5. MSC-Based Therapy of Asthma

Epidemiological data show that bronchial asthma affects approximately 300 million people worldwide [49]. In susceptible individuals, chronic airway inflammation causes recurrent episodes of airflow obstruction and bronchial hyperresponsiveness, which may lead to permanent structural changes. Thus, understanding the pathophysiology of this chronic inflammatory lung disease is essential to determining the potential applications of MSCs as an antiasthmatic therapy.

Patients suffering from atopic asthma have a genetic predisposition for the development of an antigen-specific, immunoglobulin (Ig) E-mediated response to common aeroallergens (pollen, house dust mite, and fungal spores) which is mediated by innate (DCs, mast cells, basophils, and eosinophils) and adaptive immunity cells (CD4+Th2 cells and B lymphocytes) [50]. Lung DCs capture aeroallergens, bring them to regional lymph nodes, present them to allergen-specific naive CD4+T cells, and induce generation of IL-4-, IL-5-, and IL-13-producing effector Th2 cells [51]. CD4+Th2 cells in a IL-4-dependent manner induce generation and secretion of allergen-specific IgE in plasma cells which binds to its high-affinity receptor (Fc*ε*RI), expressed on basophils and mast cells [52]. Re-exposure to the allergen causes crosslinking of Fc*ε*RI resulting in the massive release of histamine, prostaglandins, and leukotrienes from activated mast cells and basophils, which induce contraction of airway smooth muscle cells and airflow obstruction. Additionally, activated mast cells and basophils release inflammatory cytokines (TNF-*α*, IL-1*β*, IL-4, and IL-6) and chemokines enabling massive accumulation of circulating eosinophils, neutrophils, and CD4+Th2 cells in the inflamed lungs [53]. CD4+Th2 cells in an IL-5-dependent manner promote activation of eosinophils while in an IL-13-dependent manner induce goblet cell metaplasia and airway hyperresponsiveness [54]. Cytokines and matrix-degrading enzymes released from activated eosinophils and neutrophils lay the foundation for airway hyperresponsiveness and airway remodeling by causing damage to epithelial layers, promoting bronchoconstriction and deposition of extracellular matrices [50].

MSCs are able to suppress proliferation and effector function of CD4+Th2 cells, IgE production in plasma cells, and IgE-dependent activation of mast cells *in vitro* [5]. In line with these findings, several research groups demonstrated that MSCs managed to attenuate airway inflammation and remodeling and improve lung function of asthmatic animals [55–58]. Anti-inflammatory effects elicited by MSCs were mostly mediated by MSC-derived soluble factors. Cruz and coworkers suggested that MSCs altered the phenotype of antigen-specific CD4 T cells in a model of airway allergic inflammation via MSC-derived exosomes: nanosized extracellular vesicles that deliver proteins, lipids, DNA fragments, and microRNA to the target cells—immune cells, endothelial cells, pericytes, and other tissue-resident cells [55]. Similar conclusions were made by Du and colleagues who confirmed that MSC-derived exosomes alleviated airway inflammation and asthma, enhanced proliferation and immunosuppressive properties of Tregs, and enhanced production of anti-inflammatory cytokines (IL-10 and TGF-*β*) in peripheral blood mononuclear cells obtained from asthmatic patients [59].

**Figure 2:**
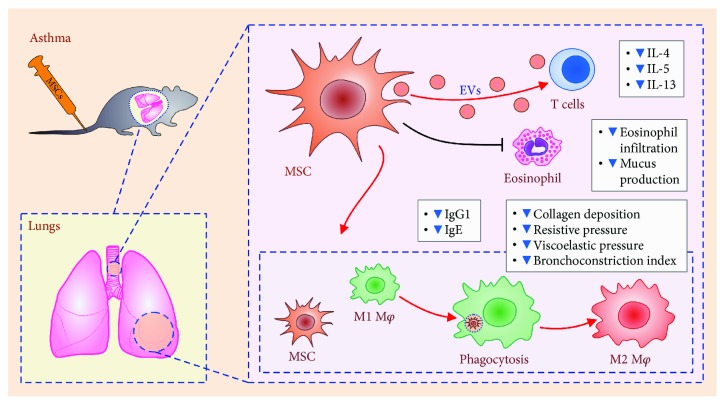
Therapeutic effects of intravenously injected MSCs in an animal model of asthma. Reduced deposition of collagen and lower bronchoconstrictive index accompanied with reduced resistive and viscoelastic pressures were noticed in MSC-treated asthmatic animals. Transplanted MSCs altered the phenotype of antigen-specific CD4 T cells in asthmatic animals via MSC-derived extracellular vesicles (EVs). Additionally, MSCs reduced eosinophil infiltration and mucus production in the lungs and downregulated levels of Th2 cytokines (IL-4, IL-5, and IL-13) in bronchial lavage, as well as serum levels of IgG1 and IgE. Alveolar macrophages become alternatively activated and developed an anti-inflammatory and immunosuppressive M2 phenotype after phagocytosis of transplanted MSCs.

In an allergic, Th2-dominant microenvironment, IL-4 and/or IL-13 activate the STAT6 pathway in the transplanted MSCs resulting in an increase in the production of TGF-*β*, which, together with Tregs (expanded by the MSCs-derived heme oxygenase-1), suppress ongoing Th2 cell-driven inflammation in the lungs [56, 60]. Intravenously injected MSCs reduced eosinophil infiltration and mucus production in the lungs and downregulated the levels of Th2 cytokines (IL-4, IL-5, and IL-13) in bronchial lavage and serum levels of IgG1 and immunoglobulin (Ig)E [56] (Figure 2).

Zeng and colleagues demonstrated that beneficial effects of MSCs in a murine model of bronchial asthma were a consequence of MSC-mediated suppression of lung myeloid DCs [61]. DCs obtained from MSC-treated asthmatic mice were immature with attenuated capacity for antigen presentation and activation of naïve T cells. Additionally, DCs from mice that received MSCs were not able to optimally migrate to the regional lymph nodes and were not able to produce an appropriate amount of chemokine ligand (CCL) 17 and CCL22 that are crucially involved in migration of effector Th2 cell in the inflamed lungs. Consequently, reduced number of IL-4-, IL-5-, and IL-13-producing Th2 cells, accompanied with downregulated serum levels of IgE, lower number of lung-infiltrated eosinophils, and reduced production of mucus were observed in MSC-treated asthmatic mice. These MSC-mediated effects resulted in significant attenuation of pulmonary inflammation, reduction of bronchial hyperresponsiveness, and notably improved lung function of MSC-treated asthmatic mice [61].

Braza and coworkers described an additional mechanism involved in MSC-mediated attenuation of bronchial asthma that was relied on the interaction between MSCs and alveolar macrophages in the injured lungs [62]. In particular, they suggested that alveolar macrophages become alternatively activated and developed an anti-inflammatory and immunosuppressive M2 phenotype after phagocytosis of transplanted MSCs [62]. Consequently, M2 macrophage produces immunosuppressive factors that suppress ongoing inflammation and promote repair and regeneration in the asthmatic lungs. In line with these findings, it was recently highlighted by Kitoko and colleagues that a cross-talk between murine BM-MSCs or AT-MSCs with alveolar macrophages is crucially important for reduced lung inflammation, airway hyporesponsiveness, and mucus hyposecretion in MSC-treated asthmatic mice and that BM-MSC or AT-MSC-mediated expansion of Tregs is not an obligatory effect of transplanted MSCs in asthmatic animals [63]. Kitoko and coworkers also concluded that pretreatment of asthmatic mice with murine BM-MSCs or AT-MSCs can increase the presence of Tregs in the lungs, while BM-MSCs and AT-MSCs were not able to induce the expansion of Tregs when lymphocytes were already allergenically primed indicating that the MSC : Treg interaction was not crucially involved in therapeutic effects of MSCs in asthma [63]. On the contrary, Li and associates noticed that human PL-derived MSCs (PL-MSCs) improved airway hyperresponsiveness and inflammation in asthmatic rats primarily by increasing the total number of IL-10-producing Tregs in the lungs which was followed by a reduced presence of lung infiltrated Th17 cells, macrophages, neutrophils, and eosinophils [64]. These, on first sight, opposite findings regarding the importance of the MSC : Treg interplay in MSC-dependent attenuation of asthma could be explained by the fact that MSCs from different sources have differential effects on immune cells [65] and that murine and human MSCs use different molecular mechanisms for generation and expansion of Tregs [5].

In addition to anti-inflammatory effects, MSC treatment prevents airway remodeling in asthmatic animals [57, 58]. Significantly reduced deposition of collagen in lung parenchyma and decreased resistive and viscoelastic pressure, accompanied with a downregulated bronchoconstriction index, were noticed in MSC-treated asthmatic mice compared to asthmatic animals that did not receive MSCs [57, 58]. Additionally, MSCs managed to reduce generation of reactive oxygen and nitrogen species responsible for oxidative stress in asthma. Transplantation of human BM-MSCs had a beneficial effect on oxidative stress, reduced the levels of nitrotyrosine and maintained the oxidative balance in asthmatic lungs of experimental animals [66].

Phosphoinositide 3-kinase (PI3K) and Notch signaling were proposed as the main molecular targets of MSCs in asthmatic lungs [67, 68]. Transplantation of MSCs affected PI3K signaling by preventing the expression of Akt phosphorylation resulting in suppressed lung inflammation and airway remodeling in asthmatic rats [67]. Reduced Notch-1, Notch-2, and jagged-1 and increased Notch-3, Notch-4, and delta-like ligand (delta)-4 expression were observed in lungs of asthmatic rats that received human PL-MSCs [68]. Alterations in the expression of the Notch signaling pathway were accompanied by polarization of immune response towards Th1 immunity as manifested by increased serum levels of IFN-*γ* and decreased serum levels of IL-4 and IgE. Furthermore, decreased goblet cell hyperplasia and mucus production were noticed in lung tissues of PL-MSC-treated asthmatic rats, indicating that MSCs suppressed asthma symptoms by modulating Notch signaling [68].

Currently, two ongoing clinical studies are planning to test the safety and efficacy of MSC-based therapy for the treatment of asthmatic patients. A phase 1 investigation will be performed at the University of Miami Miller School of Medicine with the aim of testing the safety of allogeneic BM-MSCs in the therapy of mild asthma. BM-MSCs will be delivered via peripheral intravenous infusion to 6 asthmatic patients who will be randomized in two experimental groups and will receive either 20 million or 100 million of BM-MSCs. Pulmonary function tests, lung volumes, and dyspnea questionnaires will be assessed every 4 weeks while unwanted side effects will be monitored continuously until study completion (NCT03137199).

Having in mind that MSC-based beneficial effects in asthma are mainly a consequence of MSC-derived factors, researchers from the Punta Pacifica Hospital of Panama City decided to elucidate safety and efficacy of allogeneic UC-MSC-derived trophic factors (MTF) in adult asthmatic patients. Although the study is still recruiting patients, it is planned that each of the 20 patients will intranasally receive MTF once per week for a period of 4 weeks. Side effects as well as alterations of lung function will be monitored during the one-month follow-up (NCT02192736).

## 6. Usage of MSCs in Cell-Based Therapy of COPD

COPD is characterized by persistent respiratory symptoms and airflow limitation consequent to destruction of terminal bronchioles (obstructive bronchiolitis) and lung parenchyma (emphysema), usually caused by significant exposure to noxious particles or gases [69]. The main risk factors for the development of this serious public health issue are cigarette smoking, airway hyperresponsiveness, a family history of asthma, and respiratory infections in childhood [70]. An altered function of lung-infiltrated immune cells, oxidative stress, and imbalance in activity of proteases and their inhibitors are responsible for the development of main pathological changes observed in COPD patients: progressive and persistent airflow limitation associated with an enhanced chronic inflammatory response in the airways and the lungs [71]. Due to their capacity to suppress detrimental immune response, maintain oxidative balance, and regulate activity of matrix-degrading enzymes, MSCs are considered as promising tools for cell-based therapy of COPD. Several experimental and clinical studies demonstrated beneficial effects of MSCs in the treatment of COPD [72, 73].

Intravenous, intratracheal, and intrabronchial administration of BM-MSCs and AT-MSCs (in a minimum number of 5 × 10^4^ cells/animal) was a safe therapeutic approach that showed beneficial effects in both structural and functional outcomes in the COPD animal models, which were prepared either by elastase instillation or by cigarette smoke exposure [72]. The best effects were noticed after intratracheal injection of BM-MSCs which were even superior than lung tissue-derived MSCs (LT-MSCs). Interestingly, intravenous injection of LT-MSCs resulted in immediate death of the recipient mice, a phenomenon that was not observed after intravenous administration of BM-MSC or AT-MSCs [73].

Intravenous and intratracheal injected MSCs migrated and successfully engrafted into the COPD-injured lungs of experimental animals within 24 hours after administration [74–77]. MSC transplantation significantly attenuated emphysematous changes in experimental animals as demonstrated by reduced alveolar damage and reduced alveoli number loss [78, 79]. A statistically improved pulmonary function (determined by the analysis of vital capacity (VC), forced expiratory volumen at 100 milliseconds (FEV100), dynamic compliance (Cdyn), and mean forced expiratory flow) was noticed in MSC-treated COPD animal models [72]. Improvement in histological outcomes and pulmonary function were accompanied by reduced presence of inflammatory cells in the alveolar septa and peribronchiolar and perivascular interstitium [79, 80].

**Figure 3:**
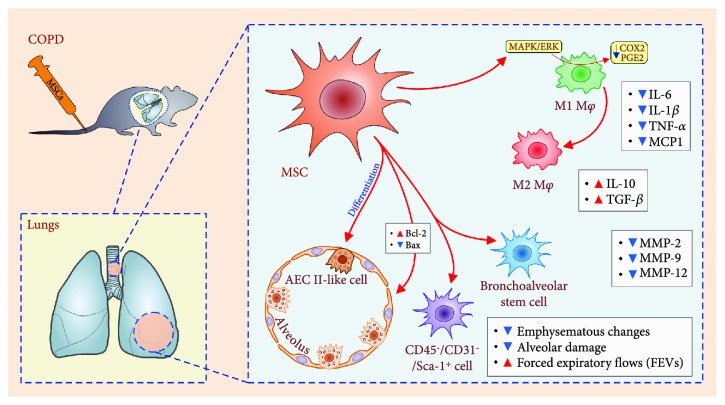
Molecular and cellular mechanisms responsible for beneficial effects of MSCs in the therapy of COPD. Reduced emphysematous changes and alveolar damage, accompanied with increased FEVs, were noticed in MSC-treated COPD animals. MSC-dependent downregulation of the COX2/PGE_2_ pathway in inflammatory M1 macrophages occurs via the p38 MAPKs and ERK pathways and resulted in macrophage polarization toward an anti-inflammatory M2 phenotype. Transplantation of MSCs significantly improved the lung architecture of COPD animals by decreasing the production of macrophage-derived MMP-2, MMP-9, and MMP-12. Additionally, transplanted MSCs either directly (through the differentiation into ATII-like cells) or indirectly (by inducing proliferation and differentiation of lung resident (CD45-/CD31-/Sca-1+) stem cells) regenerated injured lungs.

Among lung-infiltrated immune cells, the main cellular targets of transplanted MSCs in COPD animals were macrophages [75, 81] (Figure 3). MSCs, in a paracrine manner, through the production of IL-10, TGF-*β*, and HGF, suppressed cyclooxygenase-2 (COX2) expression and PGE_2_ production in alveolar macrophages [75]. MSC-mediated downregulation of the COX2/PGE_2_ pathway in inflammatory M1 macrophages occurs via the p38 mitogen-activated protein kinases (MAPKs) and extracellular signal-regulated kinase (ERK) and resulted in macrophage polarization toward an anti-inflammatory M2 phenotype [75, 82]. Accordingly, a lower expression of M1 macrophage-derived inflammatory mediators (TNF-*α*, IL-1*β*, IL-6, and monocyte chemoattractant protein 1 (MCP-1)) and a higher expression of M2 macrophage-derived anti-inflammatory IL-10 and TGF-*β* cytokines were observed in COPD animals that received MSCs [72, 75, 82]. Furthermore, transplantation of MSCs significantly improved lung architecture of COPD animals by decreasing the production of macrophage-derived MMP-2, MMP-9, and MMP-12 that mediated the degradation of elastin connective fibers in lung parenchyma and caused tissue remodeling [79].

The beneficial effect of MSCs in COPD has been also attributed to the inhibition of alveolar cell apoptosis. An inhibition of ATII cell apoptosis in MSC-treated COPD mice was a consequence of a significantly reduced expression of proapoptotic Bax and enhanced expression of the antiapoptotic Bcl-2 gene [83]. A decrease in the number of apoptotic cells was also associated with MSC-induced suppression of caspase 3, a crucial mediator of programmed cell death in ATII cells [84].

Beneficial effects of MSCs in COPD are mainly due to paracrine effects involved in the suppression of inflammation and apoptosis in the lung tissue but also may be a consequence of their differentiation into the structural cells of the alveolar unit (Figure 3) [83, 85, 86]. Liu and colleagues demonstrated that transplanted MSCs successfully engrafted into emphysematous lung tissue and were able to differentiate *in vitro* into functional, SPC-expressing ATII-like cells through the activation of the canonical Wnt/*β*-catenin pathway [85]. In line with these results, Zheng and coworkers demonstrated that transplanted MSCs successfully engrafted in the lungs, differentiate in ATII-like cells, and protected against pulmonary emphysema [83]. Additionally, MSC-based therapy of COPD mice resulted in proliferation of lung resident stem cells that represent a valuable cellular source for the replacement of injured ATII cells. The total number of endogenous stem cells (CD45-/CD31-/Sca-1+ cells) and significantly improved lung function were noticed in MSC-treated COPD mice [87]. Although these results are encouraging, it has to be emphasized that MSC-based attenuation of COPD was mainly based on the effects of MSC-derived soluble factors and that signaling pathways responsible for differentiation of MSCs in functional ATII-like cells *in vivo* need to be defined in future studies.

The only completed clinical trial which investigated the therapeutic effects of MSCs in COPD was performed in the United States (NCT00683722). Sixty-two patients with moderate to severe COPD were randomized to intravenously receive either infusion of ex vivo cultured allogeneic human MSCs (Prochymal, Osiris Therapeutics Inc.) or vehicle control. Patients received four monthly infusions (100 million MSCs/infusion) and were subsequently followed for 2 years after the first infusion [88]. Serious adverse events, increased frequency of COPD exacerbations, or worsening of disease were not observed in COPD patients treated with MSCs suggesting that systemic application of allogeneic MSCs was a safe procedure. Although downregulated serum levels of C-reactive protein in the MSC-treated group of COPD patients indicated that MSC-based therapy managed to, at least partially, suppress ongoing inflammation, pulmonary function testing as well as quality of life indicators were not significantly different between MSC-treated and nontreated COPD patients [88]. Despite the fact that obtained results were discouraging, the most important conclusion of this study was that allogeneic MSCs could be safely intravenously administrated in patients with moderate to severe COPD [88].

## 7. MSC-Based Therapy of IPF

An initiating trigger of IPF is still unclear. Recurrent lung injury, an increased apoptosis of ATII cells, aberrant epithelial-mesenchymal interactions, altered coagulation and detrimental immune response associated with enhanced fibroblast proliferation, and excessive deposition of the extracellular matrix are the main pathological changes observed in patients suffering from IPF [89–95]. Destruction of normal lung architecture leads to the development of progressive fibrosis which results in reduced pulmonary function, manifested by dry cough, dyspnea, and fatigue [96].

Since MSCs may differentiate into ATII cells in vitro, suppress production of degrading enzymes, and inhibit secretion of profibrotic factors in lung-infiltrated immune cells, several experimental and clinical studies investigated therapeutic effects of MSCs in the treatment of IPF [97–103].

Administration of MSCs prevented irradiation-induced lung fibrosis [97]. A diminished inflammatory injury in MSC-treated animals correlated with the attenuated production of inflammatory cytokines, impaired proliferation of fibroblasts, and reduced accumulation of collagen [97].

In a similar manner, transplantation of MSCs (with a dosage ranging between 0.1 × 10^6^ and 4 × 10^6^ cells) protects against bleomycin-induced lung injury and fibrosis as manifested by substantial improvement in histopathology, attenuated lung inflammation, reduced pulmonary edema, diminished collagen deposition, impaired MMP-2, MMP-9, and MMP-13 activation, and notably decreased mortality of MSC-treated animals [98]. MSCs managed to successfully engraft in bleomycin-injured lungs within 4 hours after injection. Transplanted MSCs suppressed production of nitric oxide, inflammatory cytokines (TNF-*α*, IL-1*β*, and IL-6), and profibrotic TGF-*β* in lung infiltrated immune cells and resident macrophages [99, 100]. Ortiz and coworkers managed to characterize a specific subpopulation of MSCs that express interleukin 1 receptor antagonist (IL-1Ra). These MSCs were able to, in an IL-1Ra-dependent manner, completely attenuate inflammation and pulmonary fibrosis in bleomycin-injured mice [101]. When MSC-derived IL-1Ra was bound to the IL-1 receptor (IL-1R), various proinflammatory events, initiated by IL-1 : IL-1R binding, become inhibited (including the synthesis and releases of inflammatory cytokines and chemokines accompanied with enhanced influx of neutrophils, macrophages, and lymphocytes in injured lungs), which consequently resulted with the attenuation of inflammation and fibrosis [101]. In line with these results are our findings related to the therapeutic potential of “Exosomes d-MAPPS,” whose activity was based on PL-MSC-derived exosomes containing IL-1Ra and several other imunomodulatory cytokines and chemokines (IL-27, CXCL14, and CXCL16) which are involved in immunomodulation of the immune response in the injured lungs [102]. Results, obtained in a pilot trial with a small number of patients, revealed notably attenuated lung inflammation and significantly improved pulmonary function parameters in exosome d-MAPPS-treated patients with chronic lung inflammation [102]. Similar results, related to the efficacy of MSC-derived exosomes in the therapy of lung injury and fibrosis, were obtained by Tan and coworkers who found that AF-MSC-derived exosomes attenuated fibrosis, recovered pulmonary function, and enhanced endogenous lung repair [103].

Importantly, despite the fact that MSCs can be used for the attenuation of chronic lung inflammation and fibrosis, plenty of evidence suggests that aberrant activation of Wnt/*β*-catenin and TGF-*β* signaling pathways in lung-resident MSCs might induce their differentiation towards myofibroblasts and could, consequently, contribute to the development of IPF [104]. In line with these findings, a pharmacological inhibitor of Wnt/*β*-catenin signaling (ICG-001) managed to prevent MSC-myofibroblst transition and protected from bleomycin-induced fibrosis [105]. In a similar manner, under hypoxic conditions, microRNA-145- (miR-145-) dependent inhibition of TGF-*β* receptor II (TGF-*β*RII) managed to prevent unwanted TGF-*β*-driven differentiation of MSCs into fibroblast-like cells [106].

Safety and efficacy of MSC-based therapy of IPF patients have been evaluated in several, already completed, clinical trials [107–110]. A phase 1b single-centre, nonrandomized study, which was conducted in Australia, investigated therapeutic potential of PL-MSCs that were intravenously injected in IPF patients (NCT01385644). From a total number of 8 PL-MSC-treated patients, half of them received 1 million PL-MSCs/kg bw while 4 others received 2 million PL-MSCs/kg bw. Although both doses of PL-MSCs were well tolerated, with only minor and transient alterations in peri-infusion hemodynamics and gas exchange, PL-MSC-based therapy did not result in attenuation of IPF. MSC-treated patients were followed for six months, and none of the monitored parameters (forced vital capacity (FVC), diffusing lung capacity for carbon monoxide (DLCO), six-minute walk test (6MWT), or computed tomography (CT) fibrosis score) were significantly changed by intravenous infusion of PL-MSCs [107].

Safety issues related to the MSC-based therapy of IPF were also analyzed in another phase 1 clinical trial in which 9 patients with mild to moderate IPF intravenously received 20, 100, or 200 million allogeneic MSCs (NCT02013700). None of treatment-emergent serious side effects (nonfatal pulmonary embolism, stroke, hospitalization for worsening dyspnea, and clinically significant laboratory test abnormalities) were reported. Nevertheless, during the 60 weeks of follow-up, two MSC-treated patients died because of IPF progression (disease worsening and/or acute exacerbation) and a total number of 21 adverse effects were reported (the most frequently recorded were bronchitis (in 3 patients) and common cold (in 2 patients)) [108].

More optimistic results were obtained in another phase 1b clinical study that noticed a notable improvement in quality-of-life parameters after endobronchial administration of AT-MSCs in 14 IPF patients [109]. Importantly, the recently published longitudinal outcomes of this study demonstrated that endobronchial transplantation of AT-MSCs was a safe therapeutic approach since no serious side effects (including exacerbation of IPF) were noticed in MSC-treated patients, two years after the first administration of MSCs [110]. Nevertheless, a significant functional decline occurred at 24 months after the first administration of AT-MSCs, indicating that new therapeutic strategies are urgently needed in order to prolong the therapeutic effects of transplanted MSCs.

## 8. Strategies to Enhance the Survival of Transplanted MSCs in the Injured Lungs

The beneficial effects of MSCs are relied on their capacity to home, engraft, and survive in the injured lung tissue [111]. Accordingly, optimization of MSC culture conditions has been used as an important strategy for the enhanced engraftment and prolonged survival of MSCs within the inflammatory microenvironment of the lungs. Several research groups highlighted that induction of autophagy as well as overexpression of growth factors and their receptors in MSCs may increase survival and therapeutic potential of MSCs [112–117]. It was well documented that hypoxia could induce autophagy in MSCs allowing them better survival in the inflammatory microenvironment [112]. Overexpression of hypoxia-inducible factor-1 alpha (HIF-1*α*) in MSCs significantly enhanced survival of engrafted MSCs and remarkably increased their therapeutic effects in the *Escherichia coli* model of bacterial pneumonia [113]. Since hypoxia increases production of anti-inflammatory and antifibrotic factors in MSCs, hypoxia-preconditioned MSCs managed to efficiently attenuate bleomycin-induced pulmonary fibrosis [114]. Additionally, Chen and coworkers found that ischemic postconditioning pretreatment significantly increased VEGF production in MSCs enhancing their beneficial effects in ischemia/reperfusion- (I/R-) induced lung injury [115].

It is well known that the interaction between growth factors and their receptors promotes activation of antiapoptotic pathways enabling cell survival and proliferation. Preconditioning of MSCs with TGF-*β*1 or oncostatin M (OSM) significantly increased the expression of prosurvival and antiapoptotic genes in MSCs [116, 117]. Accordingly, compared to OSM-nonprimed MSCs, OSM-preconditioned MSCs better survived and more efficiently improved the respiratory function in bleomycin-induced lung fibrotic mice [117].

## 9. Challenges towards Clinical Use of MSCs in the Therapy of Inflammatory Lung Diseases

The safety and efficacy of transplanted MSCs in the attenuation of inflammatory lung diseases seem to be reasonably proven in experimental models. However, results obtained in already conducted clinical trials pointed at several challenges which have to be addressed before MSCs will be routinely used in clinical settings.

First, some of the patients who received MSCs within a short time frame developed infection and reported respiratory symptoms indicating that MSC-based treatment resulted in excessive suppression of immune response in the injured lungs [108]. Accordingly, an optimal number of transplanted MSCs should be clearly defined with the aim of finding the right balance between their beneficial and undesired effects which could happen due to immunosuppression.

Second, therapeutic effects of autologous MSCs transplanted in the IPF patients should be carefully monitored since BM-MSCs obtained from IPF patients are senescent with significant differences in mitochondrial function, increased accumulation of DNA damage, and reduced capacity for migration and immunomodulation when compared with BM-MSCs derived from age-matched healthy subjects [118].

Third, the local microenvironment in which MSCs engraft contains factors that could induce unwanted differentiation of transplanted MSCs [119]. Therefore, new research studies should be focused in definition of factors and signaling pathways that are responsible for the fate of MSCs after their *in vivo* administration.

Finally, MSCs, in a paracrine and endocrine manner, may promote tumor growth and metastasis by suppressing antitumor immunity and by inducing neovascularization through the production of proangiogenic factors (VEGF, HGF, platelet-derived growth factor, angiopoietin-1, and placental growth factor). Accordingly, studies which utilize MSCs should be focused in continuous monitoring and long-term follow-up of MSC-treated patients in order to determine possible protumorigenic effects of MSC-based therapy [119].

## 10. Conclusions

Results obtained in a large number of preclinical studies showed that MSCs may suppress detrimental immune response in the lungs and are able to differentiate into functional ATII-like cells resulting in the attenuation of ARDS, acute lung injury, asthma, COPD, and IPF. Several already conducted clinical trials suggest that administration of MSCs were well tolerated and that MSC-based therapy is a safe therapeutic approach since only a limited number of side effects (mainly related to the MSC-based excessive suppression of immune response in the injured lungs) were reported. Accordingly, it can be concluded that, due to their potent immunomodulatory and regenerative properties, MSCs represent new therapeutic agents in the cell-based therapy of inflammatory lung diseases.

Nevertheless, considering the fact that transplanted MSCs may differentiate in undesired cell types and may promote tumor growth and metastasis in an endocrine manner, future studies must be focused on resolving these safety issues before MSCs could be offered as a new human remedy for the treatment of inflammatory lung diseases.
